# Association Between Heart Failure Ejection Fraction Phenotypes and Contrast-Associated Acute Kidney Injury: A Systematic Review

**DOI:** 10.7759/cureus.106839

**Published:** 2026-04-11

**Authors:** Kaandeeban Mohanraj, John Amerson, Naveen Punchayil Narayanankutty

**Affiliations:** 1 Internal Medicine, Indira Gandhi Medical College and Research Institute, Puducherry, IND; 2 Nephrology, University of South Florida Morsani College of Medicine, Tampa, USA

**Keywords:** acute kidney injury, contrast-associated acute kidney injury, contrast-induced nephropathy, ejection fraction (ef), heart failure, left ventricular function, meta-analysis, systematic review

## Abstract

Contrast-associated acute kidney injury (CA-AKI) is more common in patients with heart failure (HF). Stratified by left ventricular ejection fraction (LVEF), the effect of disease severity is still unknown. There is little and inconsistent data comparing the risk of CA-AKI among the various forms of HF identified by the latest guidelines of the European Society of Cardiology (ESC).

A systematic search of PubMed, Embase, Cochrane Library, and Web of Science (January 1, 2011 - December 11, 2025) for studies reporting CA-AKI outcomes stratified by LVEF phenotypes (heart failure with reduced ejection fraction (HFrEF), heart failure with mildly reduced ejection fraction (HFmrEF), and heart failure with preserved ejection fraction (HFpEF)) was performed. We planned a meta-analysis, but due to heterogeneity in adjusted models and limited study data available, we present a narrative synthesis of adjusted effect estimates. Risk of bias was assessed using Risk Of Bias In Non-randomized Studies - of Exposures (ROBINS-E).

Two observational cohort studies comprising 3,499 patients with HF met the eligibility criteria. Both studies reported multivariable-adjusted odds ratios. One study found no significant association between reduced ejection fraction (EF) and CA-AKI after adjustment (HFrEF vs. HFpEF adjusted OR 1.01, 95% CI 0.69-1.74; HFmrEF vs. HFpEF adjusted OR 1.31, 95% CI 0.87-1.96). Another study reported adjusted estimates suggesting higher odds with HFrEF compared with other phenotypes (reported adjusted OR 0.85, 95% CI 0.73-0.98 for phenotype-level comparisons). Because of the differences in study design, populations, and covariate adjustment, we did not pool estimates quantitatively; instead, we describe the findings narratively.

Current evidence is limited to two observational cohorts and yields inconsistent adjusted estimates. Therefore, pooled effect estimates weren't reported. The findings suggest that EF alone may not fully reflect kidney vulnerability. Due to the limited number of observational studies, these findings should be interpreted with caution, highlighting the need for larger studies in the future.

## Introduction and background

In modern medical practice, radiological investigations play a pivotal role in diagnostic evaluation and therapeutic decision-making. Contrast-associated acute kidney injury (CA-AKI) is associated with the use of contrast media, despite the fact that it greatly improves diagnostic accuracy. According to Kidney Disease: Improving Global Outcomes (KDIGO), it is defined as an increase in serum creatinine exceeding 25% from baseline or an absolute increase of 0.5 mg/dL, usually within 24 to 48 hours after the radiological procedure [1].

Additionally, with an incidence of more than 2% in the general population, it has been identified as one of the common causes of treatment-associated acute kidney injury, ranking third in the United States. Depending on the risk factor profile of the individuals under study, the incidence of CI-AKI after coronary angiography (CA) and percutaneous coronary intervention (PCI) ranges from 2.8% to 13% in certain studies. Serum creatinine typically peaks between two and five days and returns to baseline within approximately 14 days in most cases. The combination of renal vasoconstriction, medullary ischemia and hypoxia, oxidative stress, and direct tubular toxicity of contrast agents is theorized to drive the pathophysiology [2-4].

The reported incidence of CA-AKI varies across geographic regions and clinical settings, reflecting differences in patient characteristics, comorbidity burden, and contrast use practices, with limited standardized global estimates available. The risk of CA-AKI is substantially higher (ranging between 20 and 30%) in vulnerable populations, including patients with chronic kidney disease (CKD), advanced age, diabetes mellitus, heart failure (HF), female sex, peripheral vascular disease, and hypertension. With HF being one of the high risk groups of CA-AKI, the significance of HF phenotypes based on left ventricular ejection fraction (LVEF) has not been thoroughly examined, underscoring the clinical importance of identifying phenotype-specific risk [2-4].

Current classifications differentiate between heart failure with reduced ejection fraction (HFrEF; LVEF<40%), heart failure with mildly reduced ejection fraction (HFmrEF; LVEF 40-49%), and heart failure with preserved ejection fraction (HFpEF; LVEF≥50%). The evidence comparing the risk of CA-AKI among these groups is limited and heterogeneous [5,6]. Additionally, although several risk prediction models exist to estimate CA-AKI risk prior to contrast exposure, they do not adequately account for HF phenotypes, limiting their utility in clinical decision-making [2,3]. Due to the significant long-term complications and morbidity linked to both HF and CA-AKI, it is important to identify high-risk subgroups for prevention and risk management strategies.

In this high-risk population, determining whether LVEF-based HF phenotypes affect CA-AKI risk may enhance pre-procedural risk assessment and guide contrast-related therapeutic decision-making. The purpose of this review was to analyze the available data regarding the relationship between LVEF-based HF phenotypes and the risk of CA-AKI and related outcomes.

## Review

Materials and methods

The Preferred Reporting Items for Systematic Reviews and Meta-Analyses (PRISMA) statements guidelines and the Cochrane Collaboration Handbook for Systematic Reviews of Interventions' methodological standards were followed in the design, execution, and reporting of this systematic review [7,8]. Ethics approval was not necessary because the study is based on findings from previously published, peer-reviewed literature. The International Prospective Register of Systematic Reviews (PROSPERO) registration number for this systematic review is CRD420261301651.

Search Strategy

A thorough literature search was carried out in PubMed, Embase, the Cochrane Library, and Web of Science for studies published from January 1, 2011, to December 11, 2025. The search strategy employed Boolean operators (AND, OR) to amalgamate free-text keywords related to CA-AKI and HF with controlled vocabulary (including MeSH and Emtree terms).

The PubMed search strategy was as follows:

("contrast-induced nephropathy"[MeSH] OR "acute kidney injury"[MeSH] OR "contrast induced nephropathy"[tiab] OR "contrast-associated acute kidney injury"[tiab] OR "contrast-induced acute kidney injury"[tiab] OR "CI-AKI"[tiab] OR "contrast media"[tiab]) AND ("heart failure"[MeSH] OR "cardiac failure"[tiab] OR "heart decompensation"[tiab]) AND ("ejection fraction"[tiab] OR "left ventricular ejection fraction"[tiab] OR LVEF[tiab] OR HFrEF[tiab] OR HFpEF[tiab] OR HFmrEF[tiab]).

For the other databases (Embase, Cochrane Library, and Web of Science), search strategies were adapted from the PubMed strategy using database-specific syntax and controlled vocabulary (e.g., Emtree terms). However, full reproducible search strings for the other databases were not retained, which is acknowledged as a limitation. There were no language limits on the literature search. We also manually checked the reference lists of the studies that were included and the relevant reviews to find articles that the electronic search didn't find.

The studies were included if they enrolled adult patients with HF who underwent contrast-based diagnostic or interventional procedures and if they stratified the outcomes of contrast-associated nephropathy according to the updated European Society of Cardiology (ESC) guidelines based on LVEF, categorizing them into three groups: HFrEF (LVEF<40%), HFmrEF (LVEF 40-49%), and HFpEF (LVEF≥50%) [9] (Table 1).

**Table 1:** Eligibility criteria based on the PICO(S) framework HFrEF, heart failure with reduced ejection fraction; HFmrEF, heart failure with mildly reduced ejection fraction; HFpEF, heart failure with preserved ejection fraction; KDIGO: Kidney Disease: Improving Global Outcomes; CA-AKI, contrast-associated acute kidney injury; LVEF, Left ventricular ejection fraction.

Component	Description
Population (P)	Adult patients (≥18 years) with heart failure who underwent contrast-enhanced diagnostic or interventional procedures.
Intervention/Exposure (I)	Heart failure phenotypes stratified by LVEF: HFrEF: LVEF<40% HFmrEF: LVEF 40-49% HFpEF: LVEF≥50%
Comparator (C)	Comparison between different heart failure phenotypes, including: HFrEF vs HFpEF; HFrEF vs HFmrEF; HFmrEF vs HFpEF
Outcome (O)	Primary outcome: Incidence of CA-AKI based on study-defined criteria (e.g., KDIGO or contrast-induced nephropathy definitions). Secondary outcomes (if reported): In-hospital or long-term mortality, and requirement for renal replacement therapy
Study Design (S)	Observational studies (prospective or retrospective cohort studies, registry-based studies); Randomized controlled trials were included if they reported CA-AKI stratified by LVEF. Case reports, case series, reviews, editorials, conference abstracts without full text, pediatric studies, and animal studies were excluded.

In order to filter and eliminate duplication, all of the records obtained from the electronic database searches were exported to Rayyan (Rayyan Systems Inc., Cambridge, MA, US) [10]. Titles and abstracts were screened to find potentially eligible studies after duplicate records were eliminated. The eligibility of full texts of articles that were judged relevant was then evaluated. 

Data Extraction

A standardized data extraction form was used to extract data from studies that qualified. Study characteristics (author, year, country, study design), sample size, patient demographics, LVEF-based HF classification, definition of CA-AKI, incidence of CA-AKI, and reported effect estimates were among the variables that were extracted.

Two reviewers independently completed each step of the study selection and data extraction process; disagreements were resolved through discussion, and if unresolved, adjudicated by a third reviewer using predefined consensus criteria.

Risk of Bias Assessment

The Risk Of Bias In Non-randomized Studies - of Exposures (ROBIN-E) was used to evaluate the risk of bias for the included nonrandomized studies. The Risk-of-Bias Visualization (ROBVIS) web application was used to create plots for these [11]. Independent risk of bias assessments were carried out, and disagreements were settled by dialogue.

Data Synthesis and Statistical Analysis

We planned a meta-analysis to pool adjusted effect estimates if data from eligible studies were sufficiently homogeneous. During full-text screening, we found only two eligible studies with different adjustment sets and designs and without shared individual participant data. Because of such limitations, quantitative pooling was not performed. Instead, we attempted to present a narrative synthesis of adjusted estimates, organized by study and by comparison between different phenotypes of HF presented within each included study. We report the adjusted odds ratios (ORs) and 95% confidence intervals (CIs) as presented by the original authors and discuss differences in models and potential sources of bias. To explore the possibility of harmonized analyses, we contacted the corresponding author of one included study to request additional data; however, it was not available for sharing. Figures were generated from the adjusted effect estimates reported by the primary studies. We reproduced study-level adjusted ORs (and 95% CIs) as journal-style forest plots for clarity, and these are not pooled meta-analytic figures. These figures were adapted from the original analyses reported by the primary studies in accordance with their Creative Commons Attribution-NonCommercial 4.0 (CC BY-NC 4.0) licenses, with appropriate citations of the original sources.

Assessment of Publication Bias

Because there were only a few studies in the review, it wasn't possible to check for publication bias.

Results

The methodical search yielded 301 records from four databases. Two hundred and fifty-nine of the 261 remaining records were removed during the title and abstract screening, and 40 duplicates were discovered and removed after exporting to Rayyan. Two full-text articles were assessed for eligibility using the PICO criteria. The final systematic review included two observational cohort studies that met the inclusion criteria following eligibility evaluation.

**Figure 1:**
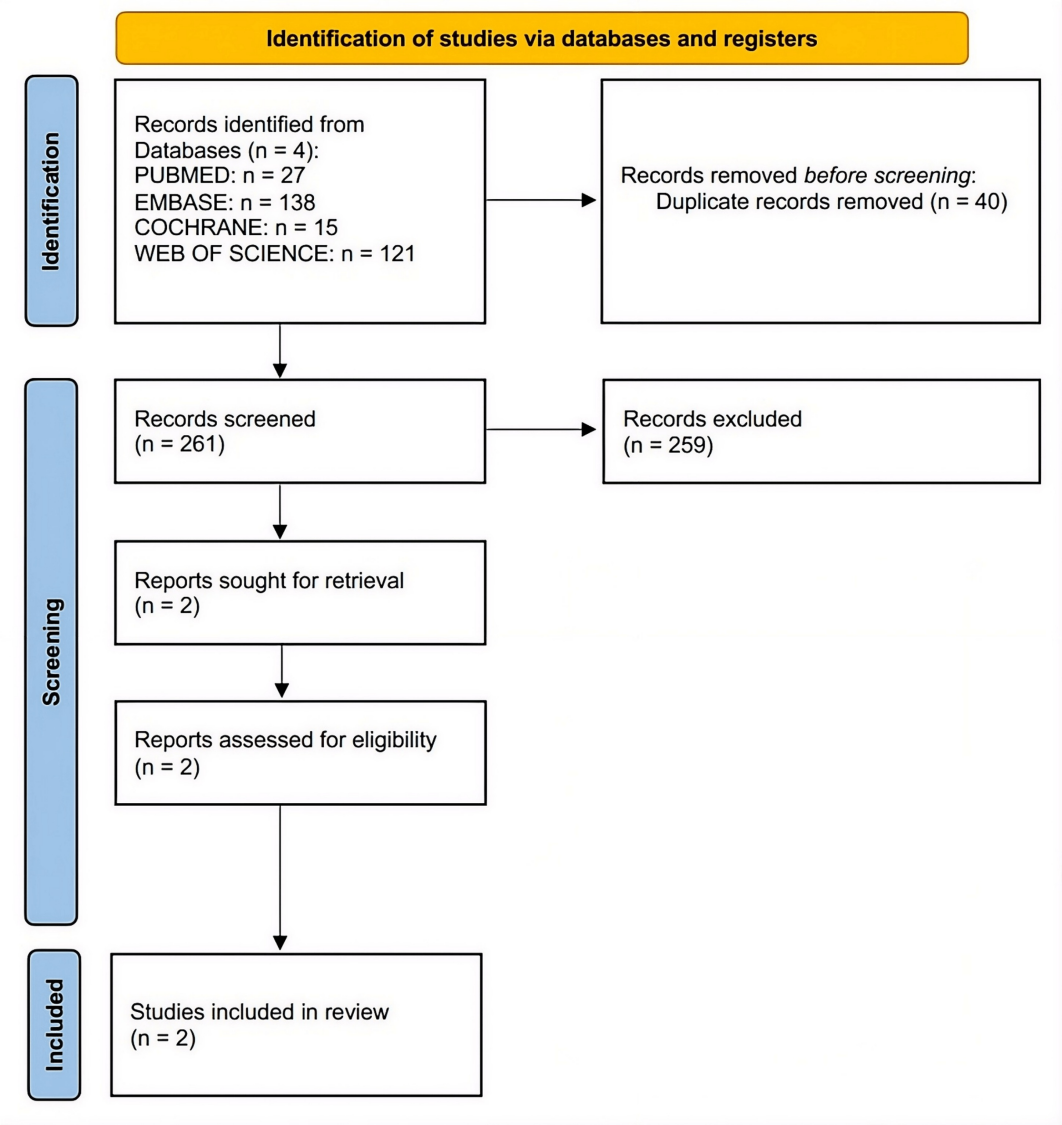
PRISMA flow diagram illustrating study selection for contrast-associated acute kidney injury in heart failure phenotypes PRISMA: Preferred Reporting Items for Systematic Reviews and Meta-Analyses

The PRISMA flow diagram, which illustrates the study selection process, is shown in Figure 1.

Risk of Bias Assessment

**Figure 2:**
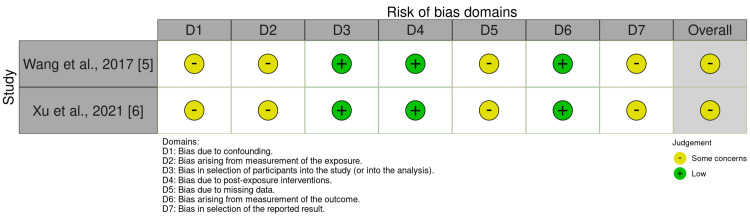
Evaluation of included observational studies for risk of bias applying ROBINS-E The ROBINS-E (Risk Of Bias In Non-randomized Studies of Exposures) tool was used to evaluate the risk of bias for the included observational cohort studies. Bias resulting from confounding, participant selection, exposure classification, deviations from intended exposure, missing data, outcome measurement, and choice of reported outcomes are among the domain-level assessments across studies that are shown in the figure. Due to possible residual confounding that is a feature of observational study designs, the overall risk of bias was deemed to have some concerns [5,6,11].

The ROBINS-E tool [11] was used to assess the risk of bias for the two observational studies, and the results showed an overall risk of bias of some concerns. Figure 2 with the ROBINS-E traffic-light plot shows a domain-level evaluation of risk of bias.

Regardless of multivariate adjustment of some clinically significant confounders, the evaluation of the results was primarily influenced by potential residual confounding and limitations related to observational designs. Furthermore, neither study included a prospectively registered analysis plan, raising some questions about selective reporting that could skew results and compromise the validity of the conclusions.

Study Characteristics

Two observational cohort studies met inclusion criteria. Both the studies were conducted in China and comprised adult patients with HF undergoing contrast-based coronary procedures. Overall, a total of 3,499 patients with HF were included across the two studies [5,6].

A prospective observational cohort of adults with HF undergoing CA or PCI was described by Wang et al. (2017) [5]. The study included 1,647 patients in total. Based on the LEVF, patients were divided into three groups with HF phenotypes: HFrEF, HFmrEF, and HFpEF. A rise in serum creatinine of at least 25% or 0.5 mg/dL from baseline measured within 48 to 72 hours after contrast media exposure was consistently used to define contrast-associated nephropathy. According to the study, 225 patients (13.7%) experienced CA-AKI. The incidence of CA-AKI among HF phenotypes was found to be 21.8%, 18.4%, and 11.2% for HFmrEF, HFrEF, and HFpEF, respectively. Clinical covariates such as age, hypertension, diabetes mellitus, renal insufficiency, advanced HF status, prior myocardial infarction, emergency PCI, contrast volume, hypotension, and specific medications were adjusted for using multivariable logistic regression models [5].

A total of 3,848 adults undergoing CA with or without PCI were included in the large multicenter retrospective observational study by Xu et al. (2021) [6]. Of these, 1,852 had HF, with a demonstrated CA-AKI incidence of 361 (19.5%). Adjusted logistic regression models were used to assess the development of CA-AKI after patients were divided into three groups according to the ESC guidelines based on the ejection fraction (EF) cutoffs. The incidence of CA-AKI data for each subgroup was not reported, despite the study reporting a total of 421, 397, and 1034 patients across the three groups HFrEF, HFmrEF, and HFpEF, respectively. Within each HF phenotype, the study also investigated relationships between EF severity and CA-AKI risk [6].

Baseline characteristics of the included studies are summarized in Table 2.

**Table 2:** Characteristics of included studies with HF stratified as HFrEF, HFmrEF, HFpEF and outcomes of contrast associated nephropathy HF, heart failure; HFrEF, heart failure reduced ejection fraction; HFmrEF, heart failure with mildly reduced ejection fraction; HFpEF, heart failure with preserved ejection fraction; CA, coronary angiography; PCI, percutaneous coronary intervention; CA-AKI, contrast-associated acute kidney injury.

Author, year	Study design	Population (n)	HF phenotype	Procedure	CA-AKI definition	Key findings
Wang et al., 2017 [5]	Prospective observational cohort	1,647 HF patients	HFrEF, HFmrEF, HFpEF	CA / PCI	≥25% or ≥0.5 mg/dL increase in serum creatinine within 48–72 h	Higher crude CA-AKI rates in HFrEF and HFmrEF; phenotype not independently predictive after adjustment
Xu et al., 2021 [6]	Retrospective observational cohort	1,852 HF patients	HFrEF, HFmrEF, HFpEF	CA ± PCI	≥25% or ≥0.5 mg/dL increase in serum creatinine within 72 h	CA-AKI incidence was 361 (19.5%); Higher odds of CI-AKI in patients with HFrEF; Lower levels of EF in HFrEF and HFmrEF associated with higher odds of CA-AKI.

Narrative Synthesis of Adjusted Estimates

Primary outcome of CA-AKI across phenotypes: Wang et al., 2017 [5] used multivariable logistic regression to assess the association between HF phenotype and the risk of CA-AKI. Reduced EF and CA-AKI risk did not show a statistically significant correlation after adjusting for clinical covariates. The adjusted odds ratios for HFrEF and HFmrEF were 1.01 (95% CI 0.64-1.60) and 1.31 (95% CI 0.84-2.05), respectively, when compared to patients with HFpEF. After taking clinical confounders into consideration, these findings suggest that variations in EF phenotype alone may not be independently linked to CA-AKI risk [5].

**Figure 3:**
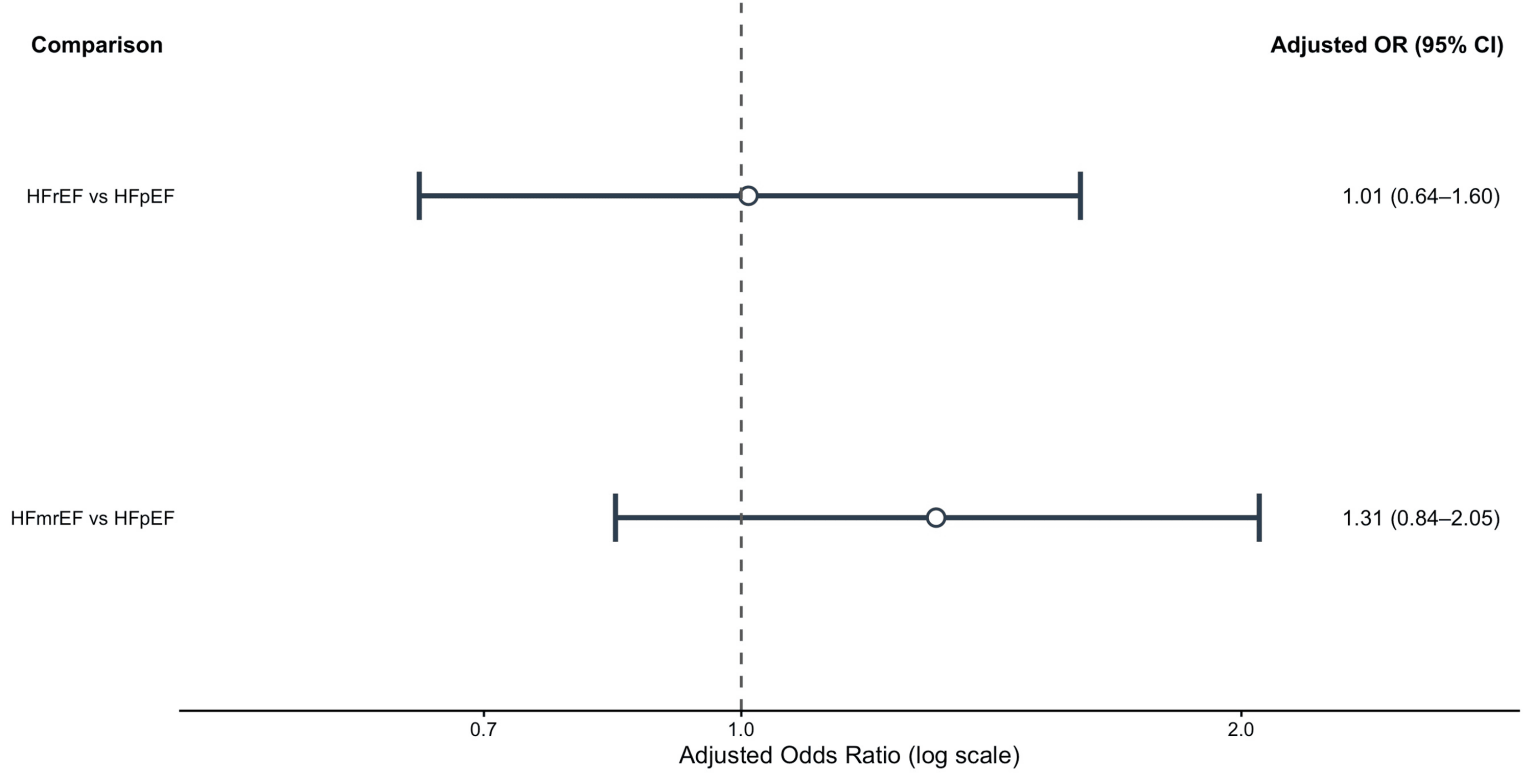
Contrast-associated acute kidney injury adjusted odds ratios for all heart failure phenotypes Adjusted odds ratios (OR) for contrast-associated acute kidney injury (CA-AKI) between heart failure with reduced ejection fraction (HFrEF) and heart failure with preserved ejection fraction (HFpEF) and heart failure with mildly reduced ejection fraction (HFmrEF) and HFpEF are displayed in a forest plot. Adjusted ORs are shown by circles, and 95% confidence intervals (CI) are shown by horizontal lines. The null value (OR=1) is shown by the dashed vertical line. The Creative Commons Attribution–NonCommercial 4.0 (CC BY-NC 4.0) license was used to adapt from Wang et al., 2017 [5].

Figure 3 shows the adjusted effect estimates that Wang et al. [5] reported.

Xu et al. [6] reported adjusted effect estimates comparing HFrEF with other HF phenotypes and looked at CA-AKI risk across HF phenotypes. The comparison of HFmrEF/HFpEF with HFrEF in the adjusted analysis produced an OR of 0.85 (95% CI 0.73-0.98), suggesting that patients with HFrEF had greater odds of CI-AKI than those with the other HF phenotypes. Compared to the other HF phenotypes in the analysis, this estimate suggested a possible higher risk of CA-AKI among patients with HFrEF [6].

Within-phenotype EF severity analysis: Within each HF phenotype subgroup, Xu et al. [6] assessed the association between EF severity and CA-AKI risk. According to the adjusted estimates, CA-AKI risk was linked to decreasing EF in both the HFrEF (OR 0.956, 95% CI 0.935-0.976; p-value <0.001) and HFmrEF (OR 0.913, 95% CI 0.870-0.957, p-value <0.001) groups, but not in the HFpEF group [6].

**Figure 4:**
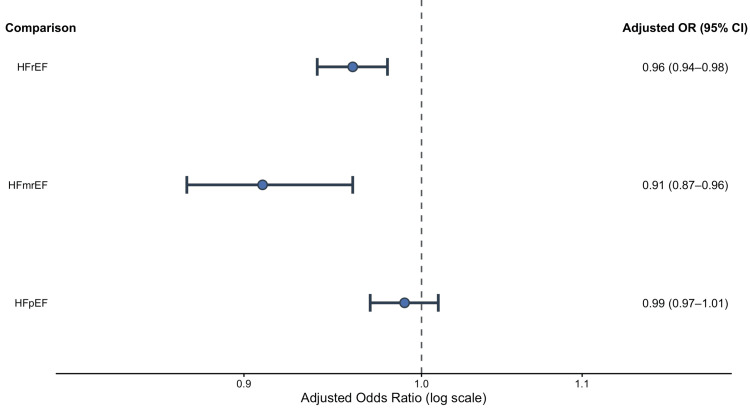
Association between ejection fraction and contrast-associated acute kidney injury within heart failure phenotypes Forest plot showing the association between ejection fraction (EF) and the risk of contrast-associated acute kidney injury (CA-AKI) within heart failure phenotypes. Points represent adjusted odds ratios (OR) and horizontal lines indicate 95% confidence intervals (CI). The dashed vertical line represents the null value (OR=1). Adapted from Xu et al., 2021 [6] under the Creative Commons Attribution–NonCommercial 4.0 (CC BY-NC 4.0) license. HFrEF, heart failure with reduced ejection fraction; HFmrEF, heart failure with mildly reduced ejection fraction; HFpEF, heart failure with preserved ejection fraction.

These adjusted associations are visualized in Figure 4.

Summary of Evidence

Overall, the two included studies reported heterogeneous adjusted estimates regarding the relationship between HF EF phenotypes and CA-AKI risk. While Wang et al. (2017) [5] found no significant association between EF phenotype and CA-AKI after adjustment, Xu et al. [6] reported a modest association in phenotype-level analysis.

Beyond CA-AKI, Wang et al. (2017) also reported clinically significant outcomes. Patients in the HFmrEF group had lower rates of hypotension (11.1%) and in-hospital mortality (5.8%) than those in the HFrEF group. Additionally, higher use of renal replacement therapy and intra-aortic balloon pump support was linked to lower EF, indicating increased hemodynamic instability in patients with lower EF. HFrEF was found to be an independent predictor of mortality in adjusted analyses (adjusted hazard ratio=2.88, 95% CI 1.77-4.69; p-value <0.001) [5].

According to Wang et al. (2017), advanced HF with Killip class >1 or New York Heart Association (NYHA) class > 2 was a significant independent risk factor (adjusted OR= 1.54, 95% CI, 1.07-2.22; P=0.019). These results imply that a major factor influencing CA-AKI risk in this population may be the clinical severity of HF rather than EF alone [5].

On the other hand, Xu et al. (2021) found that N-terminal pro-B-type natriuretic peptide (NT-proBNP) was an independent predictor of CA-AKI for all HF phenotypes, with consistent effects seen in HFrEF (OR 1.118, 95% CI 1.061-1.178; p-value <0.001), HFmrEF (OR 1.110, 95% CI 1.046-1.177; p=0.001), and HFpEF (OR 1.140, 95% CI 1.095-1.186; p-value <0.001) [6].

The results are presented through narrative synthesis because quantitative pooling of effect estimates was not carried out, due to variations in study design, populations, adjustment strategies, and the lack of subgroup data in one of the studies. Variability across studies arose from both clinical differences and methodological variation, and the direction of effect was therefore interpreted qualitatively across studies.

**Figure 5:**
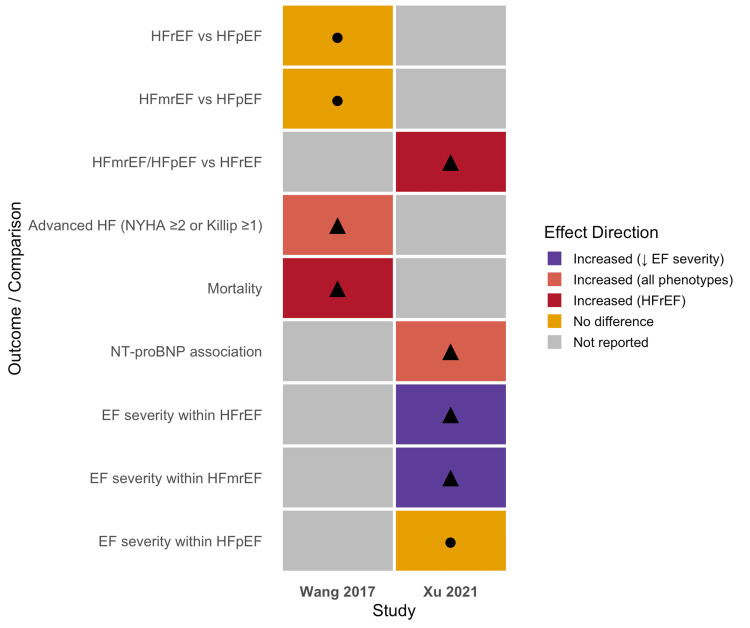
Evidence matrix of heart failure phenotypes and CA-AKI outcomes Evidence matrix summarizing the direction of associations reported across included studies evaluating contrast-associated acute kidney injury (CA-AKI) among heart failure phenotypes. Colored cells and symbols are used to represent the direction of association reported in each study, including no difference, increased risk across all phenotypes, or increased risk limited to specific phenotypes [5,6]. HFrEF, heart failure with reduced ejection fraction; HFmrEF, heart failure with mildly reduced ejection fraction; HFpEF, heart failure with preserved ejection fraction; NYHA, New York Heart Association; NT-proBNP: N-terminal pro-B-type natriuretic peptide.

An evidence matrix highlighting the connections between HF phenotypes, clinical severity markers, and CA-AKI outcomes was created in order to summarize the direction of associations reported across the included studies. The matrix offers a graphic summary of the results from the two studies that are part of this review (Figure 5).

Discussion

Although prior studies, including systematic reviews, have examined CA-AKI in HF, limited evidence is available synthesizing outcomes stratified by the HF phenotypes (HFrEF, HFmrEF, and HFpEF) defined by the updated ESC guidelines. In this systematic review, only two observational cohort studies met the inclusion criteria. The available evidence demonstrated heterogeneous adjusted estimates regarding the relationship between HF phenotypes and CA-AKI risk. These results should be viewed as exploratory rather than confirmatory due to the small number of studies and study-level differences.

While Wang et al. (2017) [5] reported no statistically significant association between EF phenotype and CA-AKI after multivariable adjustment, Xu et al. (2021) [6] reported a modest association in a phenotype-level analysis. These findings suggest that the relationship between EF phenotype and CA-AKI risk remains uncertain and may be influenced by other clinical factors and biomarkers beyond EF alone.

Although the pathophysiology of the development of contrast-associated nephropathy is not fully established, it is believed to have multiple interacting mechanisms. When hemodynamic deterioration leads to HF, it lowers blood flow to the kidneys and activates the renin-angiotensin system and the sympathetic nervous system. This leads to increased release of inflammatory mediators and oxidative stress, which contribute to the development of CA-AKI [3].

Reduced EF is associated with hemodynamic instability and impaired organ perfusion, contributing to renal vulnerability. In addition, contrast media-related factors, including increased viscosity and direct endothelial and tubular cytotoxicity, further exacerbate ischemic and inflammatory injury, culminating in CA-AKI [3].

LVEF has an important role in the evaluation of how the heart functions as well as in guiding the therapeutic decisions in patients with hemodynamic compromise and a cascade leading to decreased renal perfusion [12]. However, the role of LVEF with CA-AKI remains controversial.

In contrast, the current systematic review focused on patients who already have HF and categorized them by their EF phenotypes outlined by the ESC guidelines. In this context, the available evidence suggests that the EF phenotype alone may not be a dominant determinant of CA-AKI risk once HF is already present. The change in risk seen after adjusting emphasizes the importance of these other factors and points out the weakness of using EF alone as a risk factor for CA-AKI.

Reduced LVEF has frequently been identified as a risk factor for CA-AKI in earlier research. Nevertheless, a large number of these studies primarily contrasted patients with a lower EF with those who had normal heart function or no HF [13-15]. The review of existing literature shows a past report from a randomized trial conducted in 2010 that put forward that low EF is linked with contrast-associated nephropathy [13]. One of the recent studies conducted in Indonesia assessed the pre- and post-angiography creatinine values in patients with HFrEF and HFpEF and revealed that the mean creatinine value was higher in the HFrEF population [16].

Another study reported that in 138 patients diagnosed with acute myocardial infarction, following the second step of staged CA, the patients with low EF (≤35%) had an increase in incidence of CA-AKI (from 2% to 8%) [14]. Patients with acute HF had a two-fold risk of developing CA-AKI following CA along with an increased risk of mortality, supporting ideas of one of the included studies in this review [5,17].

Lower EF was found to be one of the strong independent predictors of AKI in 386 patients with ST-elevated myocardial infarction (STEMI) receiving primary PCI in a retrospective observational study by Shacham et al. [16]. However, even after controlling for confounders, the findings of research by Barbieri et al. and Kurtul et al. revealed the opposite effect [18,19]. Additionally, E/E' <15 was found to be an independent risk factor for the development of CA-AKI in patients with diastolic dysfunction [20].

With the search for alternate methods to monitor the severity of HF, NT-proBNP was explored as one of the valuable biomarkers [21]. Elevated levels of this biomarker indicate the ongoing hemodynamic derangements and poor myocardial and renal perfusion contributing to the development of CA-AKI. One of the studies done by Wang et al. reported the association between CA-AKI and NT-proBNP levels, supporting the idea of it being an encouraging biomarker for patients with HF undergoing CA/PCI [6,22].

Previous studies often showed a higher risk of CA-AKI in patients with reduced EF by comparing them with patients without HF. In contrast, our analysis compared different types of HF alone. These findings may explain why the difference in CA-AKI risk between the EF groups became much smaller after adjustment. 

This systematic review has several limitations. First, only two studies met the eligibility criteria based on standardized HF phenotypes. This limits the strength and generalizability of the available evidence, and the findings should be interpreted as exploratory. In addition, both included studies were observational in design, and residual confounding cannot be excluded despite the use of multivariable-adjusted estimates.

Third, the studies used different covariate adjustment strategies, preventing harmonized quantitative synthesis of adjusted estimates. Additionally, subgroup-specific CA-AKI incidence data were not available in one of the included studies, which further limited comparative analyses. Finally, the limited number of studies precluded assessment of publication bias and prevented more detailed subgroup analyses. Therefore, rather than conclusive effect estimation, the main contribution of this analysis is the identification of a clinically significant evidence gap.

## Conclusions

The evidence that is currently available indicates that the relationship between the EF phenotype of HF and the risk of CA-AKI is complex and probably influenced by a number of clinical factors other than EF, such as hemodynamic instability, neurohormonal activation, and biomarkers like NT-proBNP. Definitive conclusions cannot be drawn about the independent role of the EF phenotype in predicting CA-AKI risk due to the small number of studies and inconsistent adjusted estimates.

Given the widespread use of contrast-based procedures in patients with HF, the current scarcity of data stratified by HF phenotype highlights an important gap in the literature. As a result, rather than being definitive, this review's conclusions should be seen as hypotheses generating. To better understand the relationship between HF phenotypes and CA-AKI risk, future well-designed, multicenter prospective studies with standardized adjustment models and thorough reporting of phenotype-specific outcomes are needed.
